# Application of intestinal microbiota in marine fish for assessing the toxicity of typical pollutants: a literature review

**DOI:** 10.7717/peerj.20248

**Published:** 2025-10-28

**Authors:** Yunzhi Feng, Haolong Xu, Guohua Xu, Dong Sun

**Affiliations:** 1 Ecological and Environmental Science and Research Institute of Zhejiang Province, Hangzhou, China; 2 Zhejiang Key Laboratory of Ecological Environmental Damage Control and Value Transformation, Hangzhou, China; 3 Key Laboratory of Marine Ecosystem Dynamics, Second Institute of Oceanography, Ministry of Natural Resources, Hangzhou, China

**Keywords:** Pollutants, Gut microbiota, Toxicity, Marine fish

## Abstract

The widespread diffusion and dilution of pollutants in the ocean lead to prolonged exposure of marine organisms to low-concentration contaminated environments, raising growing concerns about the potential risks associated with chronic low-level pollution. The gut microbiota of fish plays a pivotal role in essential physiological processes, which are critical for host health. Therefore, the key microbes in the gut could serve as valuable biomarkers for assessing the toxic effects of pollutants. This article systematically reviews the structure and functions of marine fish gut microbiota, outlines the primary methodologies for assessing gut microbiota, and highlights the impacts of typical pollutants (including petroleum hydrocarbons, antibiotics, heavy metals, and microplastic) on the composition, functionality, and metabolic activities of marine fish gut microbiota. In the future, integrating multi-technology approaches to investigate the toxic mechanisms of pollutants on gut microbiota and their biodegradation pathways will represent a pivotal direction in marine ecotoxicology research.

## Introduction

As the economy develops rapidly, environmental pollution has caused increasingly severe damage to marine ecosystems. The mortality effects of pollutants on marine organisms, such as the lethal concentration 50 (LC_50_) value, have garnered extensive attention and research. However, it is noteworthy that high-concentration pollutants typically occur only around pollution sources (Zhou & Zhang, 1989). With dispersing and diluting by ocean currents, pollutants in the environment exist at low concentrations in most cases, and would cause long-term threats to marine life across broad areas (Schwarzenbach et al., 2006; Tarigan et al., 2025). Such threats manifest in various physiological impacts, including impaired growth, development, metabolic processes, and reproductive functions of marine organisms, ultimately leading to population decline. Consequently, most traditional methods focusing solely on physicochemical detection of lethal concentrations are insufficient to fully assess the ecological risks of pollutants in marine environments. Increasing attention has been directed toward the toxicological effects of pollutants at low concentrations.

Fish occupy key roles in marine food web and have important economic value (Travers-Trolet et al., 2025). Various microbes live in the digestive tract of fish, where they play crucial roles in many physiological processes, including food digestion, nutrient absorption, and immune regulation, all of which are closely related to host health (Cerf-Bensussan & Gaboriau-Routhiau, 2010; Koch et al., 2018; Luan et al., 2023). Additionally, the gut microbiota metabolites can affect the liver and brain through the microbiota-gut-liver axis and the microbiota-gut-brain axis (Cryan et al., 2019; Morais, Schreiber & Mazmanian, 2021; Tripathi et al., 2018). The gut serves as the primary target site for most pollutants upon entering the body, where the toxic substances can disrupt the stable state of the microbial communities (Medina-Félix et al., 2023). Consequently, dynamics of gut microbiota can be used as a proxy to assess the fish health condition and the toxicity of pollutants. On the other hand, the intestinal tract harbors diverse probiotic communities capable of the biotransformation of pollutants through a variety of biocatalytic reactions, including hydrolysis, reduction, N-oxide cleavage, functional group removal, and denitrification. These microbes also enhance the host’s resistance to exogenous pollutants by regulating critical physiological processes (Claus, Guillou & Ellero-Simatos, 2016; Haiser & Turnbaugh, 2013). Research on probiotics contributes to the development of bioproducts aimed at regulating the gut microbiota of fish to improve their health (Hoseinifar et al., 2016; Wang et al., 2021, 2018). The unique conditions of marine environment results in differences in both the structure and biological activity of the metabolites synthesized by probiotics, compared to their terrestrial counterparts, thereby providing new directions for the development of bioproducts (Uniacke-Lowe et al., 2024). Considering the key role of gut microbiota to the host, the interaction between intestinal microbes and pollutants has emerged as a research focus. However, only a limited proportion of studies have addressed marine fish. Given the substantial differences between freshwater and marine environments, toxicological findings derived from freshwater fish studies should not be directly extrapolated to marine ecosystems (Jeon et al., 2010). In this review, we will make a systematic summary of the composition, function and research methods of marine fish gut microflora, as well as the effects of typical pollutants such as petroleum, antibiotics, heavy metals and microplastics on microflora. Such knowledge will provide reference and inspiration for further understanding of toxic effects and ecological risks of marine pollutants. The audience for this review includes researchers and scientists studying in marine ecotoxicology, aquatic gut microbiome studies, and marine environmental health science, particularly those focused on marine fish as a model organism.

## Survey methodology

The Web of Science (WOS) Core Collection database and Google Scholar were primarily used for bibliographic searches in this study. First, to introduce and discuss the composition and function of marine fish gut microbiota, we used the following keywords: (“marine fish” AND (gut OR intestinal) AND (microbiota OR microflora OR “intestinal microbes” OR “intestinal microorganisms”). Then, to summarize the application of marine fish gut microbiota for assessing pollutant toxicity, we added the following keywords: “pollution” OR “pollutant” OR “contamination” OR “contaminant” OR “toxicity” OR “oil” OR “petroleum” OR “antibiotics” OR “microplastics OR “nanoplastics” OR “heavy metal”. Through manual proofreading, we incorporated studies based on the following inclusion criteria: the experimental animals used in these studies were marine fish, and studies discussing the effects of pollutants on the gut microbiota, including alteration in biodiversity, function, the associated bidirectional mechanisms and so on. The included studies were subsequently categorized based on types of pollutants. We also incorporated some articles on zebrafish, an important freshwater model fish, for improving the foundational basis of our work.

## Composition and function of marine fish microbiota

The fish intestinal tract harbors a diverse microbial community comprising bacteria, archaea, fungi, viruses, and protists, with bacteria being the predominant group (Rombout et al., 2011). The intestinal microbiota of fish can be divided into two primary groups: indigenous microbes colonizing the mucosal surfaces and free-living allochthonous microbes. The resident ones predominantly consist of Proteobacteria, Firmicutes, Bacteroidetes, Fusobacteria, Actinobacteria, and Verrucomicrobia (Ringø et al., 2006). The diversity and community structure of fish intestinal microbiota are affected by many factors such as the host, sex, diet, and environment factors (Luan et al., 2023). Generally, the aerobic and facultative anaerobes from the phylum Proteobacteria are predominant in the fish gut (Rombout et al., 2011) compared to mammals like humans, primarily due to the typically higher oxygen within the fish intestinal environment (Singh et al., 2025). Significant variations of gut microbiota exist between marine fish and freshwater fish due to the distinct environment. Some marine herbivorous fish gut harbored more anaerobes from Firmicutes that aided fermentative digestion (Egerton et al., 2018). At the genus level, the gut microbiota is more distinct between marine fish and freshwater fish (Singh et al., 2025). For example, in the most common phylum Proteobacteria, freshwater fish often contain species such as *Aeromonas, Pseudomonas, and Enterobacter* (Deb, Das & Das, 2020; Givens et al., 2015), while marine fish usually harbor species such as *Vibrio*, *Photobacterium*, and *Shewanella* (Egerton et al., 2018; Givens et al., 2015).

The gut microbiota plays a crucial regulatory role in numerous key physiological processes of the fish, including nutrient absorption, development, immune defense, nervous system function, endocrinology and so on (Luan et al., 2023; Ou et al., 2021). Many bacteria genera, such as *Bacillus*, *Vibrio*, *Photobacterium*, *Aeromonas*, *Flavobacterium*, and *Pseudomonas*, may participate in the digestive process of fish gut (Ray, Ghosh & Ringø, 2012). In marine fish, several microbes found in the gut have specific functions. For instance, *Aliivibrio* are involved in bioluminescence (Klemetsen, Karlsen & Willassen, 2021) and *Pseudoalteromonas* have important function in interactions with the host (Drønen et al., 2022). However, many of these genera also contain pathogenic species (Egerton et al., 2018). The intestinal microbiome of fish is a complex and dynamic environment, where diverse microbial taxa maintain a state of dynamic equilibrium. Environmental stressors, exemplified by pollutant exposure, can disrupt the balance of the gut microbiota. Previous studies have suggested that some genera present in marine fish gut have the potential to degrade pollutants (Table 1). Theses bacterial genera were originally isolated from environmental samples and their pollutant-degrading capabilities are well-documented in these environments. However, direct evidence of their function in fish guts remains limited. In freshwater fish, some probiotic strains have been found to mitigate the intestinal toxicity of pollutants by reducing their accumulation in the gut or alleviating tissue oxidative stress and inflammation. For instance, *Lactobacillus reuteri* and *Lactobacillus plantarum* have demonstrated the capacity to alleviate the toxicity of lead (Giri et al., 2018) and cadmium (Zhai et al., 2017), respectively. Considering the strong variation between marine and freshwater environment, whether the presence and function of these species can be applied to marine fish was unknown. Research on these functional intestinal bacteria will provide fundamental support for developing biological agents and pharmaceuticals to enhance the resistance of fish to pollutants.

**Table 1:** Potential pollutant-degrading bacterial genera identified in environmental samples, with hypothesized relevance to marine fish gut microbiota.

Class	Genus	Pollutants	References related to their functions	References related to their presence in marine fish gut
Alphaproteobacteria	*Vibrio*	Polycyclic aromatic hydrocarbons	Hedlund & Staley (2001)	Egerton et al. (2018)
Alphaproteobacteria	*Thalassospira*	Polycyclic aromatic hydrocarbons	Kayama, Kanaly & Mori (2022)	Kang et al. (2021)
Betaproteobacteria	*Burkholderia*	Aromatic hydrocarbons, antibiotics, heavy metals	Denef (2007), Jiang et al. (2008)	Wang et al. (2020)
Betaproteobacteria	*Acinetobacter*	Aromatic hydrocarbons, heavy metals, alkane	Czarny et al. (2020), Révész et al. (2020)	Egerton et al. (2018)
Gammaproteobacteria	*Alteromonas*	Selenium, tellurium	Reddy, Pathak & Nancharaiah (2023)	Egerton et al. (2018)
Gammaproteobacteria	*Alcanivorax*	Alkane	Hara, Syutsubo & Harayama (2003)	Walter, Bagi & Pampanin (2019)

## Common methods used in investigating gut microbiota

### Culture-dependent methods

Early investigations of fish intestinal microbiota were predominantly carried out by conventional microbial culture methods (Ringø & Birkbeck, 1999). These approaches enabled the identification and quantification of isolated strains, thereby revealing the composition and functional characteristics of intestinal microbes. Furthermore, using diverse selective media could obtain functional intestinal bacteria with unique physiological and biochemical properties. This methodological framework is essential for validating microbial functions, determining their mechanistic roles within the intestinal ecosystem (Ceja-Navarro et al., 2015), and subsequently developing novel microbial agents to enhance fish immunity (Dawood & Koshio, 2016). Whereas, 97% of marine intestinal bacteria are still uncultured due to their unique growth environment (Soh et al., 2024), indicating the limitations of traditional culture methods. Recently, the emerging culturomics provides a new tool for studying the function and role of intestinal microorganisms. Culturomics refers to an advanced methodology that employs various culture conditions to simulate the natural growth environment of microbes, thereby facilitating the cultivation of previously unculturable bacterial species. This approach integrates matrix-assisted laser desorption/ionization time-of-flight mass spectrometry (MALDI-TOF MS) and amplicon sequencing techniques for rapid bacterial identification and optimization of culture conditions. Through this innovative strategy, a substantial number of novel bacterial strains have been successfully isolated and characterized (Lagier et al., 2018). Currently, culturomics is mainly used in the study of human intestinal microorganisms. The primary limitations of this approach reside in its requirement for extensive testing and optimization of diverse culture conditions, coupled with high research cost (Lagier et al., 2018).

### Molecular-based methods

**(1) Amplicon sequencing**

As the high-throughput sequencing and bioinformatics technology develop, the culture-independent molecular-based methods have greatly improved our understanding of fish gut microbiota. The amplicon sequencing technology has become the most common method to study the diversity and community structure of intestinal microbes (Ghanbari, Kneifel & Domig, 2015). This method enables rapid, efficient and cost-effective analysis of microbial diversity, community structure and their response to pollution in the fish gut, and enable identifying key bacteria with biomarker potential. The amplicon sequencing method is usually based on second-generation sequencing platforms such as 454 pyrosequencing and Illumina to sequence one or more variable regions of the marker gene (*e.g*., 16S rRNA for bacteria and archaea, as well as internal transcribed spacer (ITS) or 18S rRNA for fungi). However, due to the short sequencing read length of these second-generation platforms, the taxonomic identification results from sequence are usually limited to the genus or family level. The emerging third-generation sequencing platforms such as PacBio and Nanopore enable the full-length sequencing of the 16S/18S rDNA or ITS gene, and thus significantly improve the accuracy of species annotation. However, there are also problems such as higher cost and more sequencing error. Furthermore, the amplicon sequencing technology, which relies on polymerase chain reaction (PCR) amplification, is inevitably affected by methodological limitations including primer mismatch and interspecies variations in genomic copy numbers. These methodological limitations induce amplification biases, thereby compromising the accuracy of microbial diversity characterization and relative abundance quantification (Alberdi et al., 2018; Rathod & Silverman, 2025).

**(2) Meta-omics**

The rapid advancement of omics technologies has provided novel approaches for elucidating comprehensive functional profiles of gut microbial communities. Firstly, metagenomics offers significant advantages by directly performing large-scale random shotgun sequencing of the total genomic DNA extracted from samples. This methodological innovation enables accurate characterization of microbial community composition at the species level, and could effectively avoid amplification biases due to independent of primer selection and PCR amplification processes (Hugenholtz & Tyson, 2008). In addition, metagenomics can obtain the whole genomic information of the microbiota, so it can reveal the changes of functional genes in the community and identify key functional genes that are sensitive to pollutants. To further analyze the expression levels of functional genes, the metatranscriptome method is needed. Through mRNA isolation and enrichment, followed by reverse transcription into cDNA and subsequent high-throughput sequencing, transcriptomics enables the identification of active microbes within the community as well as the characterization of dynamic changes in expression levels of functional gene. This approach provides valuable insights into pollutant metabolic processes at the transcriptional level (Sorek & Cossart, 2010). When combined with metagenomics results, it is also possible to determine which bacteria in the community are transcribing and estimate the transcription rates. The primary limitations of this technology come from the inherent characteristics of mRNA, including its low abundance and susceptibility to degradation, which makes sample preparation process quite challenging. Furthermore, while high-throughput sequencing provides comprehensive profiling capabilities, it lacks precise quantitative accuracy. To obtain reliable quantification of taxonomic abundance and functional gene expression levels, complementary techniques such as quantitative real-time PCR (qPCR) and fluorescence *in situ* hybridization (FISH) are typically employed.

The information obtained from DNA/RNA is still partial, because gene transcripts can form a variety of proteins through different ways of splicing and post-translational modification (Cho, 2007). Since proteins are directly involved in multiple biological processes, understanding their types and functions are crucial for revealing the response of the organism to environmental changes. The metaproteomics approach works by separating and extracting proteins, mass spectrometry (MS) analysis, and database matching. This method helps reveal the protein expression patterns in microbial communities, providing deeper insights into both the toxic effects and the degradation mechanism of pollutants (Franzosa et al., 2015). Despite its potential, metaproteomics has limited application in fish gut microbiota research due to several challenges, including high costs, technical complexity, and the issue of host contamination (Ou et al., 2021).

Emerging after genomics, transcriptomics, and proteomics, metabolomics represents the most phenotype-proximal omics. This approach primarily employs nuclear magnetic resonance (NMR) and MS technologies to analyze the complete set of small-molecule metabolites (molecular weight <1,000 Da) within biological systems, including amino acids, sugars, vitamins, lipids, and so on (Nicholson, Lindon & Holmes, 1999). Metabolites are the end products of gene expression, subtle changes in genes and proteins may be amplified at the metabolic level. Therefore, metabolites detection techniques have the highest sensitivity for assessing functional changes. Moreover, the number of metabolites is less than that of proteins, contributing to lower technical difficulty than metaproteomics analysis. In current fish gut microbiome toxicology studies, metabolomics is typically used in conjunction with amplicon technology to analyze the interaction between gut microbiota and metabolic levels of host under pollutants (Duan et al., 2024; Wang et al., 2023; Zhang et al., 2022).

### Gnotobiotic models

Gnotobiotic models refer to animals that are raised in germ-free environments or colonized with specific microbial species (Pham et al., 2008). These models serve as precise tools for investigating the bidirectional relationship between gut microbiota and host by eliminating interference from indigenous microbes. Gnotobiotic models have been established in several marine fish species, such as Atlantic cod (*Gadus morhua* L.) (Forberg, Arukwe & Vadstein, 2011), sea bass (*Dicentrarchus labrax*) (Dierckens et al., 2009), and Atlantic salmon (*Salmo salar* L.) (Gómez de la Torre Canny et al., 2023). These models are not only utilized to investigated the interactions between host and opportunistic pathogens (Li et al., 2015), but also enable evaluation of probiotic candidates (Aerts et al., 2018; Schaeck et al., 2017) and bioproducts (Yaacob et al., 2017). Future research with fish gnotobiotic models should integrate molecular-based methods to systematically map the bidirectional pollutant-microbiota interactions. By transplanting specific gut microbes into gnotobiotic fish models, the functions and mechanisms of key bacterial strains underlying pollutant toxicity and degradation could be validated, providing a theoretical foundation for pollution management in aquaculture.

### Summary of methods

**Figure 1:**
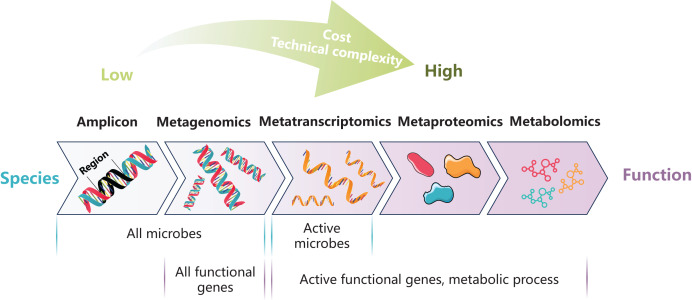
Summary of molecular-based methods for gut microbiota research.

Culture-dependent methods and molecular-based approach are both important for current research on the toxicological effects of pollutants on fish gut microbiota. In practical investigations, the selection of appropriate methodologies should be guided by specific research objectives (Fig. 1, Table 2). For instance, the amplicon sequencing with mature technology and low cost is suitable for the analysis of large-scale samples, which is helpful to establish the risk assessment system of pollutants. The multi-omics methods, which is informative but costly, is suitable for in-depth exploration of the interaction between pollution and fish gut microbiota. The challenges for future multi-omics research include enhancing the quality of sequencing data, improving the accuracy and coverage of reference databases, and integrating large amounts of data from various omics. Additionally, *in vitro* culture experiments, and gnotobiotic fish models can be used for subsequent verification. The combination of these methods can provide a theoretical basis for the derivation of environmental and health criteria for pollutants as well as the development of bioremediation.

**Table 2:** Comparison of molecular-based methods for gut microbiota research.

Methods	Objects	Advantages	Limitations
Amplicon	Regions or full-length of the marker gene	It can obtain species composition and relative abundance. **(Who are they?)**	PCR-bias.
			Taxonomic identification are usually limited to genus or family level.
Metagenomics	All genes	Taxonomic identification can be assigned to species and even strains level.	Higher sequencing throughput is required.
		It can obtain composition and relative abundance of functional genes. **(What can they do?)**	It cannot distinguish between active and dormant/dead microbes.
Metatranscriptomics	mRNA	It can obtain information of active microbes. It can identify actively expressed genes and metabolic pathways. **(What are they doing?)**	Due to low abundance and poor stability of mRNA, preparation and preservation of samples are quite challenging.
Metaproteomics	All protein	It can obtain the types and quantities of all proteins in the microbiota, and subsequent reveal protein expression patterns. **(How do they function?)**	The extraction and identification techniques are complex.
			It is difficult to eliminate host contamination.
Metabolomics	All small-molecule metabolites	It can obtain the types and quantities of all small-molecule metabolites (molecular weight < 1,000 Da), reflecting the metabolic state. **(What is the result?)**	Only small-molecule metabolites can be measured.
			It is difficult to determine the specific biological origin and action pathway of metabolites

## Effects of typical pollutants on gut microbiota of marine fish

### Petroleum pollutants

Oil spill accidents resulting from marine transportation and offshore oil extraction, coupled with substantial discharges of oil-containing wastewater, have posed significant threats of petroleum pollution to marine environments (Chen et al., 2018; Yu, Xia & Du, 2024). Upon entering the marine ecosystem, petroleum initially forms surface oil slicks that impede air-water exchange, subsequently undergoing dispersion, migration, and weathering processes driven by wind and ocean currents. This leads to widespread exposure of marine organisms to low-concentration petroleum pollutants in the water. Petroleum represents a highly complex mixture primarily composed of toxic components, such as saturated hydrocarbons, aromatic hydrocarbons, and heavy metals (Jovančićević et al., 2007). Exposure of fish intestinal systems to either crude petroleum or its secondary metabolites transformed by the liver can induce significant shifts in gut microbiota composition. This community succession is predominantly characterized by the enrichment of microbes capable of utilizing petroleum components as carbon sources (Améndola-Pimenta et al., 2020; Cerqueda-García et al., 2020; Walter, Bagi & Pampanin, 2019), which causes harm to the functional structure of gut microbiota. There are several studies focus on the response of marine fish gut microbiota to petroleum pollution as follows. Bagi et al. (2018) conduct a 28-day chronic exposure experiment to Atlantic cod by simulating the leakage scenario of low-concentration crude oil (0.01, 0.05, 0.1 mg/L). The result showed that the morphological parameters of fish in all experimental groups were not significantly different from the control group. However, the intestinal microflora of Atlantic cod exposed to medium (0.05 mg/L) and high (0.1 mg/L) concentrations of dispersed crude oil showed significant changes, reflecting the sensitivity of intestinal microflora to the toxicity of low-concentration pollutants. Specifically, the relative abundance of Deferribacteres in the gut increases with oil exposure levels, indicating it can be used as a potential biomarker of oil exposure in Atlantic cod. Spilsbury et al. (2022) fed juvenile Asian seabass (*Lates calcarifer*) with diets spiked with petroleum hydrocarbons for 33 days. They presume that the enrichment of *Photobacterium* in the intestine microbiota was proportional to the dietary exposure concentration of polycyclic aromatic hydrocarbons (PAHs). Consequently, *Photobacterium* was identified as a potential biomarker genus of PAHs exposure. Beyond amplicon sequencing, a comprehensive study using multi-omics techniques was conducted on juvenile Atlantic cod exposed to 0.05 mg/L dispersed crude oil (Magnuson et al., 2023). After 28 days of exposure, a metagenomic tool predicted significant changes in gut microbial functions, particularly affecting energy and carbohydrate metabolism, fatty acid and amino acid biosynthesis, as well as cellular structure formation. Metatranscriptomics data showed differential expression of 58 genes after 7 days of exposure. Notably, the transcriptomic response in intestinal contents was more sensitive, showing significant changes after just 1 day of exposure, reflecting the dynamic interactions between gut microbiota and host. Another study examined the effects of water accommodated fraction of a light crude oil (5% and 10% w/w) on the lined sole (*Achirus lineatus*) from the Gulf of Mexico over 28 days (Cerqueda-García et al., 2020). The research revealed an enrichment of menaquinone and demethylquinone biosynthesis pathways in the gut, suggesting these changes might shift the intestinal redox state to be more oxygen-limited. In summary, analyzing fish gut microbiota opens new possibilities for developing biomarkers of petroleum pollutant exposure. Multi-omics technologies can uncover the functional and metabolic impacts of petroleum pollutants, contributing to the construction of highly sensitive and specific toxicity assessment framework.

### Antibiotics

Antibiotics, defined as natural or synthetic compounds capable of killing bacteria or inhibiting bacterial growth, are widely used in disease treatment. However, their extensive misuse, combined with low bioavailability, high water solubility, and environmental persistence, pose significant ecological risks. When introduced into organisms, antibiotics are primarily absorbed through the intestinal tract, where they can damage intestinal tissues and eliminate substantial populations of sensitive gut microorganisms (Morgun et al., 2015; Zhou et al., 2018). Additionally, this exposure may lead to the development of antibiotic resistance within the gut microbiota (Francino, 2016). Wang et al. (2023) found that short-term (9 days) exposure to the fluoroquinolone antibiotic enrofloxacin (5–500 µg/L) increased the ratio of firmicutes abundance to Bacteroidetes abundance (F:B ratio) in the gut microbiota of marine medaka (*Oryzias melastigma*). The elevated (F:B ratio) is seen as an indicator of gut microbiota disorders (Shen et al., 2018). At the genus level, the relative abundance of several pathogenic bacteria in the genera *Flavobacteria* and *Vibrio*, as well as the probiotic bacterium *Shewanella* decreased. It reflects the imbalance of intestinal microbiota under enrofloxacin exposure. Moreover, enrofloxacin exposure led to significant enrichment of antibiotic resistance genes, as well as further negative effects on liver metabolism *via* the gut-liver axis. He et al. (2022) reported the intergenerational effect of the sulfanilamide antibiotic sulfadiazine on the intestinal flora of marine medaka. After the parents were exposed to sulfadiazine (5 mg/g) through diet for 30 days, the diversity of intestinal microbiota in the offspring was significantly reduced, and the relative abundance of Cyanobacteria was significantly increased. These intergenerational effects reflect the long-term risk of antibiotics to marine organisms.

Antibiotics are also extensively used in aquaculture for disease treatment, potentially impacting fish gut health. Sumithra et al. (2022) administered florfenicol, a chloramphenicol-class antibiotic, at a therapeutic dose of 10 mg/kg in feed to snubnose pompano (*Trachinotus blochii*) for 10 days, following by withdraw. By means of conventional culturing method, they found a 2-log reduction in the total viable bacteria compared to the control fish gut, which restored at the 10th-day post-withdrawal. The 16S amplicon sequencing approach revealed significantly reduced gut microbiota diversity, with increased relative abundance of Proteobacteria and decreased relative abundance of Firmicutes, following by their restoration at 10th-day post-withdrawal. However, the relative abundances of some specific taxa, including Vibrionaceae and Enterobacteriaceae, remained elevated compared to controls after 15th-day post-withdrawal. Additionally, the results indicated that therapeutic exposure to florfenicol neither promoted irreversible enrichment of resistant bacteria nor caused irreversible increase in antibiotic resistance in the fish gut. Sumithra et al. (2024) also evaluated the effects of oxytetracycline, another antibiotic for therapeutic use, on snubnose pompano over a 10-day exposure period. They found that the perturbation of gut microbiota recovered in 5–15 days of withdraw, without inducing antibiotic resistance. These results indicated that the toxic effects of antibiotics at a therapeutic dose on fish gut microbiota are partially reversible. In summary, dynamic monitoring of fish gut microbiota facilitates a comprehensive assessment of antibiotic exposure risks. Further studies are needed to investigate the dose-time-effect relationships of antibiotics on the gut microbiota of marine fish, and subsequently establish environmental and health-based criteria. These researches will provide scientific guidance for more rational antibiotic use in mariculture.

### Heavy metals

Heavy metal pollution has become one of the most severe global environmental challenges. Heavy metals have extremely wide sources, which not only involves natural activities such as submarine volcanic eruptions and crustal movements, but also includes human-induced leaks from industries like oil, mining, agriculture, and chemicals engineering (Abd Elnabi et al., 2023). Heavy metals have strong bioaccumulation properties. Once they enter the intestinal tract, they can damage the intestinal mucosa, alter the composition and metabolic characteristics of the intestinal flora, and thereby trigger various metabolic diseases (Duan et al., 2020). To the best of our knowledge, research on the interaction of heavy metals and the intestinal flora of marine fish is relatively limited. Liu et al. (2024) reported that after the red-spotted pufferfish (*Takifugu rubripes*) exposed to copper (50–500 μg/L) for 3 days, the relative abundances of various pathogenic bacteria in the fish gut increased, while the relative abundances of some probiotics decreased. The correlation analysis results of 16S amplicon and metabolomics suggest that the key response bacteria exposed to high copper concentration may up-regulate the level of gut metabolites in the tryptophan metabolic pathway, leading to intestinal metabolic dysbiosis. Duan et al. (2024) conducted a 14-day exposure experiment using 1 μg/L lead on the grouper (*Epinephelus fuscoguttatus*), an economically important marine fish species. The results demonstrated that lead exposure induced gut microbiota dysbiosis characterized by increased relative abundances of Proteobacteria and Actinobacteria, as well as an elevated F:B ratio. Furthermore, significant correlations were observed between the relative abundances of specific bacterial genera and hepatic physiological metabolites, suggesting that lead stress negatively affects the physiological homeostasis of *E. fuscoguttatus* through the gut-liver axis. Wang et al. (2020) investigated the response of combined exposure to cadmium (10 μg/L), lead (50 μg/L), and zinc (100 μg/L) on the gut microbiota of marine medaka for 1 month, revealing significant gender-specific differences. Compared to females, males exhibited more pronounced alterations in metabolic pathways, including carbohydrate metabolism, lipid metabolism, and biodegradation of xenobiotics. These results indicate greater sensitivity of male gut microbiota to heavy metals, potentially mediated by sex hormones (Mueller et al., 2006). However, the underlying mechanisms of gender-specific responses to heavy metal toxicity require further elucidation.

### Microplastics

Microplastics, defined as plastic particles with diameters <5 mm (Thompson et al., 2004), have been detected in marine environments worldwide, exerting a substantial threat on marine biota (Ugwu, Herrera & Gómez, 2021). These particles undergo gradual fragmentation into smaller sizes through photodegradation and weathering processes in the ocean (Ryan et al., 2009), yet resist complete degradation. Hence, microplastic pollution has emerged as one of the most pressing environmental concerns. Due to their small size, microplastics are readily ingested by fish and accumulate in their gastrointestinal tracts (Lusher, McHugh & Thompson, 2013; Norhazwani et al., 2021). Such accumulation can disrupt gut microbial communities, with the impact degree influenced by particle size and exposure periods. Kang et al. (2021) studied the toxicity of two particle sizes of polystyrene microplastics (10 µg/mL) to juvenile marine medaka after 24-h acute exposure. The 16S amplicon results revealed a significant decrease in the relative abundance of Bacteroidetes and the probiotic genus *Shewanella* in the gut, while the relative abundance of *Thalassospira*, known for its ability to degrade various PAHs (Table 1), showed a marked increase. This suggests that *Thalassospira*, may be involved in the degradation of plastics. In summary, the acute exposure to microplastics caused disruption of intestinal flora, and microplastics with a diameter of 45 μm had a greater effect than nanoplastics with a diameter of 50 nm. Gu et al. (2020) conducted a 14-day chronic exposure experiment using 100 nm polystyrene nanoplastics (5.50 × 10^−6^ – 5.50 × 10^−1^ µg/mL) on juvenile large yellow croaker (*Larimichthys crocea*). The amplicon sequence results showed that the relative abundances of Bacteroidetes and Firmicutes in the gut generally increased. While Proteobacteria showed a downward trend, indicating that the growth of juvenile fish may be inhibited by nanoplastics. At the genus level, the proportion of the potential pathogenic bacteria *Alistipes* and *Parabacteroide* increased, posing a threat of microplastics to juvenile fish health. Conversely, the rise in the probiotic *Lactobacillus* may represent a compensatory response to counteract this threat, demonstrating the self-regulatory capacity of gut microbiota in response to nanoplastic exposure. Yao et al. (2024) exposed golden pompano (*Trachinotus blochii*) to 5 μm polystyrene microplastics (10–1,000 µg/L) for 14 days. The results of amplicon sequencing and metabolomics revealed both dysbiosis and metabolic alterations (involving lipids, glucose, and amino acids). These disruptions impaired the digestion and absorption functions and may lead to many diseases of the host. Feng et al. (2021) found that after feeding 2 and 200 μm polystyrene (3 µg/mg) to marine medaka for 28 days, the carbon/nitrogen/phosphorus/sulfur metabolic pathways of gut microorganisms were changed, which is revealed by metagenomic analysis. Moreover, the strong correlation between gut microbiota dynamics and hepatic transcriptomic response demonstrated that polystyrene exposure may affect host health by disturb the gut-liver axis. Wen et al. (2024) found that after marine medaka being exposured to low concentrations of polyethylene and polylactic acid (0.2 µg/mL) for 60 days, the antibacterial *Streptomyces* was significantly enriched in the gut, but the overall community structure of intestinal flora showed no significant difference to the control group. These findings indicated that although long-term exposure to low-concentration microplastics does not induce overall gut microbiota dysbiosis, the stress response of several bacterial taxa can serve as early warning signals for chronic toxic effects. Given the persistent nature of microplastics, their long-term accumulation in the environment may exert prolonged impacts on marine organisms throughout their life cycles and even across generations. Therefore, future research should prioritize investigating the chronic toxicity of microplastics and their transgenerational effects on the gut microbiota of marine fish.

### Summary

The toxic effects of typical pollutants on marine fish gut microbiota are summarized in Table 3. Generally, these effects are manifested in two aspects as below: first, direct effects, where pollutants accumulate in the intestines, leading to changes in the composition, function, and metabolic pathways of the gut microbiota; second, indirect effects, where pollutants damage the intestinal mucosa, making microbial metabolites affect host health through the gut-liver axis or gut-brain axis. Under pollutant-induced stress, the gut microbiota can self-regulate to some extent. Various gut microbes have been found to possess the ability to mitigate the toxicity of pollutants, reducing their adverse effects on host health.

**Table 3:** Effects of typical pollutants on marine fish gut microbiota.

Types	Pollutants	Model organisms	Administered way	Main effects on gut microbiota composition	Main effects on function and metabolic profiles	References
Petroleum pollutant	Dispersed crude oil	Atlantic cod (*Gadus morhua*)	0.01, 0.05, and 0.1 mg/L; 28 d	**0.05 and 0.1 mg/L** groups: Bacteroidetes↓, Deferribacteres↑, Porphyromonadaceae↓, Rikenella↓, Ruminococcaceae↓, *Alistipes*↓, *Clostridiales↓* **0.01 mg/L** group: no significant effect	–	Bagi et al. (2018)
	Crude oil and fuel oil	Juvenile Asian seabass (*Lates calcarifer*)	1% w/w in diet; 33 d	*Photobacterium↑, Vibrio↑*	–	Spilsbury et al. (2022)
	Dispersed crude oil	Juvenile Atlantic cod	0.05 mg/L; 28 d	*Aliivibrio↑, Mycoplasma↑, Photobacterium↑*	58 differentially expressed genes related to DNA, RNA, and ATP binding, translation, signal transduction, and Wnt signaling pathway	Magnuson et al. (2023)
	Water accommodated fraction (WAF) of crude oil	Lined sole (*Achirus lineatus*)	5% and 10% w/w; 28 d	Bacteria associated to hydrocarbon degradation↑, such as *Acinetobacter johnsonii, Alcanivorax diselolei, *and *Sneathiella chungangensis*	Menaquinone and demethylquinone biosynthesis pathways↑	Cerqueda-García et al. (2020)
Antibiotics	Enrofloxacin	Marine medaka (*Oryzias melastigma*)	5 and 500 **µ**g/L; 9 d	*Escherichia↑, Epulopiscium↑, Acinetobacter↑, Apibacter↑, Vibrio↓, Halioglobus↓, Shewanella↓, Flavobacterium↓, Haliea↓*	Antibiotic resistance genes↑; Hepatic metabolism disorder associated with intestinal flora dysbiosis	Wang et al. (2023)
	Sulfadiazine	Marine medaka	5 mg/g in diet; 30 d (Parental exposure)	Verrucomicrobia↓, Cyanobacteria↑	For **female**: Carbohydrate metabolism↓ For **male**: nine pathways significantly changed (*e.g*., Carbohydrate metabolism↓, lipid metabolism↓, glycan synthesis, metabolism↑)	He et al. (2022)
	Florfenicol	Juvenile snubnose pompano (*Trachinotus blochii*)	10 mg/kg in diet; 10 d, followed by withdraw for 15 d	**Restore** after withdraw: Proteobacteria↑, Euryacrcheota↓, Firmicutes↓ *Enterovibrio↑*, *Vibrio↑*, *Pseudomonas↑*, *Shewanella↑* **Remain** after withdraw: *Serratia↓*, *Acinetobactera↓*	Kanamycin resistant microbes↑, multidrug resistance encoding genes↑ (**restored after withdraw**)	Sumithra et al. (2022)
	Oxytetracycline	Juvenile snubnose pompano	80 mg/kg in diet; 10 d, followed by withdraw for 15 d	Firmicutes↓, Actinobacteria*↓*, γ-Proteobacteria↓, *Vibrio↓, Solirubrobacteriales↑*, *Mycoplasma↑*, *Mycoplasma↓*kanamycin and ampicillin-resistant cultivable bacteria*↓* (**restored after withdraw**)	Energy metabolism pathway*↓*	Sumithra et al. (2024)
Heavy metal	Copper (Cu)	Red-spotted pufferfish (*Takifugu rubripes*)	50, 100, and 500 **µ**g/L; 3 d	**500 µg/L** group: *Granulicella*↑, Family*_XIII_AD3011_group*↓ **100 µg/L** group: *Rothia*↑, *Corynebacterium*↑, *Lactobacillus*↓, *Clostridium_sensu_stricto_1*↓ **50 µg/L** group: *Lachnoclostridium↑*, *Bergeyella ↑*, *Actinobacillus↑*, *Butyrivibrio*↓, *Ruminococcus_torques_group*↓	Significant correlation between the changes of key gut bacteria and gut metabolites related to energy and immunity (*e.g*., the arginine biosynthesis pathway and tryptophan metabolism pathway)	Liu et al. (2024)
	Lead (Pb)	Grouper (*Epinephelus fuscoguttatus*)	1 **µ**g/L; 14 d	Proteobacteria↑, Actinobacteria↑, *Streptococcus*↑, *Bacteroidales S24–7 group*↑, *Ruminococcaceae UCG-005*↑, *Ruminococcaceae UCG-014*↑, *Oscillibacter*↑, *Tenacibaculum*↓, *Bdellovibrio*↓	Functions of primary bile acid biosynthesis and glycosphingolipid biosynthesis↑; Significant correlation between the changes of key gut bacteria and the physiological indexes and metabolites in the liver	Duan et al. (2024)
	Cadmium (Cd), Lead (Pb), Zinc (Zn)	Marine medaka	Combined exposure of Cd 10 **µ**g/L, Pb 50 **µ**g/L, and Zn 100 **µ**g/L; 1 month	For **female**: Bacteroidetes↑, Verrucomicrobia↑, *Lachnoclostridium-10*↑, *Ruminococcaceae*↑, Lactobacillus↑, *Burkholderiales*↑, *Pseudomona*↑ For **male**: Cyanobacteria↑, *Aurantimonadaceae*↑, *Rhizobium*↑, *Rhizobiaceae*↑, *Paracoccus*↑, *Methylobacterium*↑, *Methylobacteriaceae*↑, *Aurantimonadacea*↑	Males exhibited more pronounced alterations in metabolic pathways than females, including carbohydrate metabolism, lipid metabolism, and biodegradation of xenobiotics	Wang et al. (2020)
Microplastics	Polystyrene (45 µm, 50 nm)	Juvenile marine medaka	10 µg/mL; 24 h	Bacteroidetes↓, *Vicingus*↓, *Shewanella*↓, *Lewinella*↑, *Pseudomonas*↑, *Thalassospira*↑, *Parahaliea*↑	–	Kang et al. (2021)
	Polystyrene (100 nm)	Juvenile large yellow croaker (*Larimichthys crocea*)	5.50 × 10^−6^, 5.50 × 10^−3^, and 5.50 × 10^−1^ µg/mL; 14 d	Bacteroidetes↑, Firmicutes↑, Proteobacteria↓, *Alistipes↑*, *Parabacteroide↑*, Lactobacillus↑	–	Gu et al. (2020)
	Polystyrene (5 µm)	Golden pompano (*Trachinotus blochii*)	10, 100, and 1,000 µg/L; 14 d	*Mycoplasma*↓	Disruptions in lipid metabolism, carbohydrate metabolism, and amino acid metabolism; activated hormones, cell growth, signaling pathways, and disease-related pathways.	Yao et al. (2024)
	Polystyrene (2 µm, 200 µm)	Marine medaka	3 µg/mg in diet; 28 d	Bacteroidetes↑, Planctomycetes↑, *Motilimonas*↓, *Propionigenium*↓, *Aliivibrio*↓, *Oleibacter*↓, *Pseudophaeobacter*↑, *Bythopirellula↑*, OM60 *[NOR5] clade↑*, *Rhodopirellula*↑, *Winogradskyella*↑, *Rubripirellula*↑, *Crocinitomix↑*, *Actibacter*↑, *Cryomorpha*	Carbon degradation/fixation activities↑, stress-related genes↑, changes of the nitrogen/phosphorus/sulfur metabolic pathways; Significant correlation between the changes of key gut bacteria and the metabolitic pathways of the liver	Feng et al. (2021)
	Polyethylene and polylactic acid	Marine medaka	0.2 µg/mL; 60 d	*Streptomyces*↑	–	Wen et al. (2024)

## Conclusions and future prospects

The microbes in marine fish exhibit various diversity and functions and plays a crucial role in maintaining host homeostasis. Multiple pollutants can exert effects on the gut microbiota, thereby impacting the health of marine fish. With advancements in multi-omics technologies, the bidirectional interactions between marine pollutants and the fish gut microbiota have been increasingly investigated. However, current toxicological studies on marine fish gut microbiota still have some limitations: (1) Research are mostly limited to descriptive analysis based on 16S amplicon sequencing results. Since fish gut microbes have significant individual variability (Clements et al., 2014), this will make it difficult to accurately attribute variation in gut microbiota to pollutants exposure (Chi et al., 2021); (2) The toxicity endpoints and safe concentrations of most marine pollutants remain largely unconfirmed up to now. Meta-omics techniques can identify toxicity endpoints more sensitive than traditional methods (Simmons et al., 2015). However, a critical gap persists in translating these sensitive indicators, such as specific microbial species or functional genes, into validated biomarkers. Current experiments based on meta-omics typically employ restricted concentration gradients. This limitation impedes the establishment of robust dose-response relationships, leaving the feasibility of these potential biomarkers uncertain.

**Figure 2:**
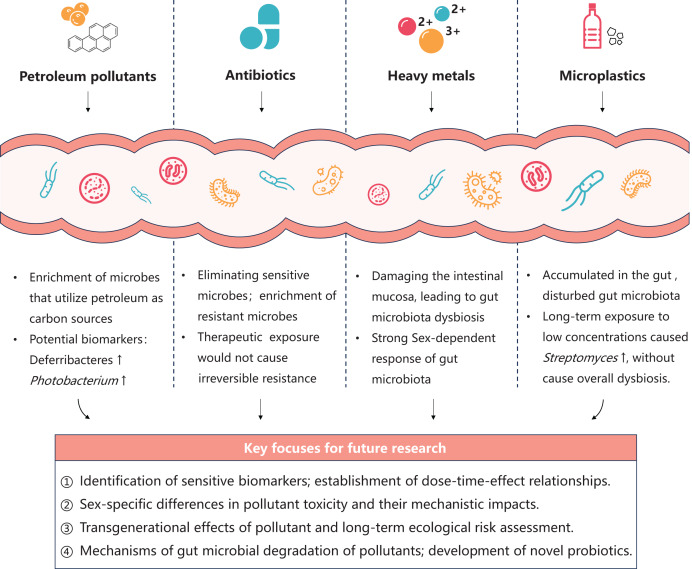
Summary of toxicity effect of typical pollutants on marine fish gut microbiota and future research perspectives.

Future research should integrate culture-based experiments, multi-omics technologies, and gnotobiotic fish models to thoroughly investigate the effects of pollutants on gut microbiota function and metabolism. Key focuses include elucidating the dose-time-effect relationships of pollutants, exploring sex-specific differences and transgenerational effects in toxicity, and developing gut probiotics capable of mitigating pollutant-induced harm (Fig. 2). These efforts will comprehensively reveal the potential impacts of pollutants on host health and marine ecosystems, while supporting sustainable practices in marine aquaculture.

## Additional Information and Declarations

### Data Availability

The following information was supplied regarding data availability:

This study is a literature review.

## References

[ref-1] Abd Elnabi MK, Elkaliny NE, Elyazied MM, Azab SH, Elkhalifa SA, Elmasry S, Mouhamed MS, Shalamesh EM, Alhorieny NA, Abd Elaty AE, Elgendy IM, Etman AE, Saad KE, Tsigkou K, Ali SS, Kornaros M, Mahmoud YAG (2023). Toxicity of heavy metals and recent advances in their removal: a review. Toxics.

[ref-2] Aerts J, Schaeck M, De Swaef E, Ampe B, Decostere A (2018). *Vibrio lentus* as a probiotic candidate lowers glucocorticoid levels in gnotobiotic sea bass larvae. Aquaculture.

[ref-3] Alberdi A, Aizpurua O, Gilbert M, Bohmann K (2018). Scrutinizing key steps for reliable metabarcoding of environmental samples. Methods in Ecology and Evolution.

[ref-4] Améndola-Pimenta M, Cerqueda-García D, Zamora-Briseño JA, Couoh-Puga D, Montero-Muñoz J, Árcega-Cabrera F, Ceja-Moreno V, Pérez-Vega JA, García-Maldonado JQ, del Río-García M, Zapata-Pérez O, Rodríguez-Canul R (2020). Toxicity evaluation and microbiota response of the lined sole *Achirus lineatus* (Chordata: Achiridae) exposed to the light petroleum water-accommodated fraction (WAF). Journal of Toxicology and Environmental Health, Part A.

[ref-5] Bagi A, Riiser ES, Molland HS, Star B, Haverkamp THA, Sydnes MO, Pampanin DM (2018). Gastrointestinal microbial community changes in Atlantic cod (*Gadus morhua*) exposed to crude oil. BMC Microbiology.

[ref-6] Ceja-Navarro JA, Vega FE, Karaoz U, Hao Z, Jenkins S, Lim HC, Kosina P, Infante F, Northen TR, Brodie EL (2015). Gut microbiota mediate caffeine detoxification in the primary insect pest of coffee. Nature Communications.

[ref-7] Cerf-Bensussan N, Gaboriau-Routhiau V (2010). The immune system and the gut microbiota: friends or foes?. Nature Reviews: Immunology.

[ref-8] Cerqueda-García D, Améndola-Pimenta M, Zamora-Briseño JA, González-Penagos CE, Árcega-Cabrera F, Ceja-Moreno V, Rodríguez-Canul R (2020). Effects of chronic exposure to water accommodated fraction (WAF) of light crude oil on gut microbiota composition of the lined sole (*Achirus lineatus*). Marine Environmental Research.

[ref-9] Chen J, Zhang W, Li S, Zhang F, Zhu Y, Huang X (2018). Identifying critical factors of oil spill in the tanker shipping industry worldwide. Journal of Cleaner Production.

[ref-10] Chi L, Tu P, Ru H, Lu K (2021). Studies of xenobiotic-induced gut microbiota dysbiosis: from correlation to mechanisms. Gut Microbes.

[ref-11] Cho WC (2007). Proteomics technologies and challenges. Genomics Proteomics Bioinformatics.

[ref-12] Claus SP, Guillou H, Ellero-Simatos S (2016). The gut microbiota: a major player in the toxicity of environmental pollutants?. NPJ Biofilms and Microbiomes.

[ref-13] Clements KD, Angert ER, Montgomery WL, Choat JH (2014). Intestinal microbiota in fishes: what’s known and what’s not. Molecular Ecology.

[ref-14] Cryan JF, O’Riordan KJ, Cowan CSM, Sandhu KV, Bastiaanssen TFS, Boehme M, Codagnone MG, Cussotto S, Fulling C, Golubeva AV, Guzzetta KE, Jaggar M, Long-Smith CM, Lyte JM, Martin JA, Molinero-Perez A, Moloney G, Morelli E, Morillas E, O’Connor R, Cruz-Pereira JS, Peterson VL, Rea K, Ritz NL, Sherwin E, Spichak S, Teichman EM, van de Wouw M, Ventura-Silva AP, Wallace-Fitzsimons SE, Hyland N, Clarke G, Dinan TG (2019). The microbiota-gut-brain axis. Physiological Reviews.

[ref-15] Czarny J, Staninska-Pięta J, Piotrowska-Cyplik A, Juzwa W, Wolniewicz A, Marecik R, Ławniczak Ł, Chrzanowski Ł (2020). *Acinetobacter* sp. as the key player in diesel oil degrading community exposed to PAHs and heavy metals. Journal of Hazardous Materials.

[ref-16] Dawood MAO, Koshio S (2016). Recent advances in the role of probiotics and prebiotics in carp aquaculture: a review. Aquaculture.

[ref-17] Deb S, Das L, Das SK (2020). Composition and functional characterization of the gut microbiome of freshwater pufferfish (*Tetraodon cutcutia*). Archives of Microbiology.

[ref-18] Denef VJ, Coenye aPV T (2007). Biodegradation of organic anthropogenic pollutants by *Burkholderia* species. Burkholderia: Molecular Microbiology and Genomics.

[ref-19] Dierckens K, Rekecki A, Laureau S, Sorgeloos P, Boon N, Van den Broeck W, Bossier P (2009). Development of a bacterial challenge test for gnotobiotic sea bass (*Dicentrarchus labrax*) larvae. Environmental Microbiology.

[ref-20] Drønen K, Roalkvam I, Nilsen H, Olsen AB, Dahle H, Wergeland H (2022). Presence and habitats of bacterial fish pathogen relatives in a marine salmon post-smolt RAS. Aquaculture Reports.

[ref-21] Duan Y, Yang Y, Zhang Z, Nan Y, Xiao M (2024). The toxic effect of lead exposure on the physiological homeostasis of grouper: insight from gut-liver axis. Marine Pollution Bulletin.

[ref-22] Duan H, Yu L, Tian F, Zhai Q, Fan L, Chen W (2020). Gut microbiota: a target for heavy metal toxicity and a probiotic protective strategy. Science of the Total Environment.

[ref-23] Egerton S, Culloty S, Whooley J, Stanton C, Ross RP (2018). The gut microbiota of marine fish. Frontiers in Microbiology.

[ref-24] Feng S, Zeng Y, Cai Z, Wu J, Chan LL, Zhu J, Zhou J (2021). Polystyrene microplastics alter the intestinal microbiota function and the hepatic metabolism status in marine medaka (*Oryzias melastigma*). Science of the Total Environment.

[ref-25] Forberg T, Arukwe A, Vadstein O (2011). A protocol and cultivation system for gnotobiotic Atlantic cod larvae (*Gadus morhua* L.) as a tool to study host microbe interactions. Aquaculture.

[ref-26] Francino MP (2016). Antibiotics and the human gut microbiome: dysbioses and accumulation of resistances. Frontiers in Microbiology.

[ref-27] Franzosa EA, Hsu T, Sirota-Madi A, Shafquat A, Abu-Ali G, Morgan XC, Huttenhower C (2015). Sequencing and beyond: integrating molecular ‘omics’ for microbial community profiling. Nature Reviews Microbiology.

[ref-28] Ghanbari M, Kneifel W, Domig KJ (2015). A new view of the fish gut microbiome: advances from next-generation sequencing. Aquaculture.

[ref-29] Giri SS, Yun S, Jun JW, Kim HJ, Kim SG, Kang JW, Kim SW, Han SJ, Sukumaran V, Park SC (2018). Therapeutic effect of intestinal autochthonous *Lactobacillus reuteri* P16 against waterborne lead toxicity in *Cyprinus carpio*. Frontiers in Immunology.

[ref-30] Givens CE, Ransom B, Bano N, Hollibaugh JT (2015). Comparison of the gut microbiomes of 12 bony fish and 3 shark species. Marine Ecology Progress Series.

[ref-32] Gómez de la Torre Canny S, Nordgård CT, Mathisen AJH, Degré Lorentsen E, Vadstein O, Bakke I (2023). A novel gnotobiotic experimental system for Atlantic salmon (*Salmo salar* L.) reveals a microbial influence on mucosal barrier function and adipose tissue accumulation during the yolk sac stage. Frontiers in Cellular and Infection Microbiology.

[ref-31] Gu H, Wang S, Wang X, Yu X, Hu M, Huang W, Wang Y (2020). Nanoplastics impair the intestinal health of the juvenile large yellow croaker *Larimichthys crocea*. Journal of Hazardous Materials.

[ref-33] Haiser HJ, Turnbaugh PJ (2013). Developing a metagenomic view of xenobiotic metabolism. Pharmacological Research.

[ref-34] Hara A, Syutsubo K, Harayama S (2003). *Alcanivorax* which prevails in oil-contaminated seawater exhibits broad substrate specificity for alkane degradation. Environmental Microbiology.

[ref-35] He S, Li D, Wang F, Zhang C, Yue C, Huang Y, Xie L, Zhang YT, Mu J (2022). Parental exposure to sulfamethazine and nanoplastics alters the gut microbial communities in the offspring of marine madaka (*Oryzias melastigma*). Journal of Hazardous Materials.

[ref-36] Hedlund BP, Staley JT (2001). *Vibrio cyclotrophicus* sp. nov., a polycyclic aromatic hydrocarbon (PAH)-degrading marine bacterium. International Journal of Systematic and Evolutionary Microbiology.

[ref-37] Hoseinifar SH, Ringø E, Shenavar Masouleh A, Esteban MÁ (2016). Probiotic, prebiotic and synbiotic supplements in sturgeon aquaculture: a review. Reviews in Aquaculture.

[ref-38] Hugenholtz P, Tyson GW (2008). Metagenomics. Nature.

[ref-39] Jeon J, Kannan K, Lim HK, Moon HB, Ra JS, Kim SD (2010). Bioaccumulation of Perfluorochemicals in Pacific oyster under different salinity gradients. Environmental Science & Technology.

[ref-40] Jiang C, Sheng X, Qian M, Wang Q (2008). Isolation and characterization of a heavy metal-resistant *Burkholderia* sp. from heavy metal-contaminated paddy field soil and its potential in promoting plant growth and heavy metal accumulation in metal-polluted soil. Chemosphere.

[ref-41] Jovančićević B, Vrvić M, Schwarzbauer J, Wehner H, Scheeder G, Vitorović D (2007). Organic-geochemical differentiation of petroleum-type pollutants and study of their fate in danube alluvial sediments and corresponding water (Pančevo Oil Refinery, Serbia). Water, Air, and Soil Pollution.

[ref-42] Kang H-M, Byeon E, Jeong H, Kim M-S, Chen Q, Lee J-S (2021). Different effects of nano- and microplastics on oxidative status and gut microbiota in the marine medaka *Oryzias melastigma*. Journal of Hazardous Materials.

[ref-43] Kayama G, Kanaly RA, Mori JF (2022). Comprehensive genomic characterization of marine bacteria *Thalassospira* spp. Provides insights into their ecological roles in aromatic hydrocarbon-exposed environments. Microbiology Spectrum.

[ref-44] Klemetsen T, Karlsen CR, Willassen NP (2021). Phylogenetic revision of the genus *Aliivibrio*: intra- and inter-species variance among clusters suggest a wider diversity of species. Frontiers in Microbiology.

[ref-45] Koch BEV, Yang S, Lamers G, Stougaard J, Spaink HP (2018). Intestinal microbiome adjusts the innate immune setpoint during colonization through negative regulation of MyD88. Nature Communications.

[ref-46] Lagier J-C, Dubourg G, Million M, Cadoret F, Bilen M, Fenollar F, Levasseur A, Rolain J-M, Fournier P-E, Raoult D (2018). Culturing the human microbiota and culturomics. Nature Reviews Microbiology.

[ref-47] Li X, Bossier P, Dierckens K, Laureau S, Defoirdt T (2015). Impact of mucin, bile salts and cholesterol on the virulence of *Vibrio anguillarum* towards gnotobiotic sea bass (*Dicentrarchus labrax*) larvae. Veterinary Microbiology.

[ref-48] Liu P, Liu Y, Cheng J, Xia Y, Yang Y (2024). Copper exposure causes alteration in the intestinal microbiota and metabolites in *Takifugu rubripes*. Ecotoxicology and Environmental Safety.

[ref-49] Luan Y, Li M, Zhou W, Yao Y, Yang Y, Zhang Z, Ringø E, Erik Olsen R, Liu Clarke J, Xie S, Mai K, Ran C, Zhou Z (2023). The fish microbiota: research progress and potential applications. Engineering.

[ref-50] Lusher AL, McHugh M, Thompson RC (2013). Occurrence of microplastics in the gastrointestinal tract of pelagic and demersal fish from the English channel. Marine Pollution Bulletin.

[ref-51] Magnuson JT, Monticelli G, Schlenk D, Bisesi JH, Pampanin DM (2023). Connecting gut microbiome changes with fish health conditions in juvenile Atlantic cod (*Gadus morhua*) exposed to dispersed crude oil. Environmental Research.

[ref-52] Medina-Félix D, Garibay-Valdez E, Vargas-Albores F, Martínez-Porchas M (2023). Fish disease and intestinal microbiota: a close and indivisible relationship. Reviews in Aquaculture.

[ref-53] Morais LH, Schreiber HL, Mazmanian SK (2021). The gut microbiota-brain axis in behaviour and brain disorders. Nature Reviews Microbiology.

[ref-54] Morgun A, Dzutsev A, Dong X, Greer RL, Sexton DJ, Ravel J, Schuster M, Hsiao W, Matzinger P, Shulzhenko N (2015). Uncovering effects of antibiotics on the host and microbiota using transkingdom gene networks. Gut.

[ref-55] Mueller S, Saunier K, Hanisch C, Norin E, Alm L, Midtvedt T, Cresci A, Silvi S, Orpianesi C, Verdenelli Maria C, Clavel T, Koebnick C, Zunft Hans-Joachim F, Doré J, Blaut M (2006). Differences in fecal microbiota in different european study populations in relation to age, gender, and country: a cross-sectional study. Applied and Environmental Microbiology.

[ref-56] Nicholson JK, Lindon JC, Holmes E (1999). ‘Metabonomics’: understanding the metabolic responses of living systems to pathophysiological stimuli via multivariate statistical analysis of biological NMR spectroscopic data. Xenobiotica.

[ref-57] Norhazwani J, Ahmad A, Syafiq MM, Mazlan M, Hafidz Yusoff A, Mat Lazim A (2021). Occurrence, distribution and characteristics of microplastics in gastrointestinal tract and gills of commercial marine fish from Malaysia. Science of the Total Environment.

[ref-58] Ou W, Yu G, Zhang Y, Mai K (2021). Recent progress in the understanding of the gut microbiota of marine fishes. Marine Life Science & Technology.

[ref-59] Pham LN, Kanther M, Semova I, Rawls JF (2008). Methods for generating and colonizing gnotobiotic zebrafish. Nature Protocols.

[ref-60] Rathod D, Silverman JD (2025). PCR bias impacts microbiome ecological analyses. BioRxiv: The Preprint Server for Biology.

[ref-61] Ray AK, Ghosh K, Ringø E (2012). Enzyme-producing bacteria isolated from fish gut: a review. Aquaculture Nutrition.

[ref-62] Reddy KKG, Pathak S, Nancharaiah YV (2023). Aerobic reduction of selenite and tellurite to elemental selenium and tellurium nanostructures by *Alteromonas* sp. under saline conditions. International Biodeterioration & Biodegradation.

[ref-67] Révész F, Figueroa-Gonzalez PA, Probst AJ, Kriszt B, Banerjee S, Szoboszlay S, Maróti G, Táncsics A (2020). Microaerobic conditions caused the overwhelming dominance of *Acinetobacter* spp. and the marginalization of *Rhodococcus* spp. in diesel fuel/crude oil mixture-amended enrichment cultures. Archives of Microbiology.

[ref-63] Ringø E, Birkbeck TH (1999). Intestinal microflora of fish larvae and fry. Aquaculture Research.

[ref-64] Ringø E, Sperstad S, Myklebust R, Refstie S, Krogdahl Å (2006). Characterisation of the microbiota associated with intestine of Atlantic cod (*Gadus morhua* L.): the effect of fish meal, standard soybean meal and a bioprocessed soybean meal. Aquaculture.

[ref-65] Rombout JH, Abelli L, Picchietti S, Scapigliati G, Kiron V (2011). Teleost intestinal immunology. Fish & Shellfish Immunology.

[ref-66] Ryan PG, Moore CJ, van Franeker JA, Moloney CL (2009). Monitoring the abundance of plastic debris in the marine environment. Philosophical Transactions of the Royal Society B: Biological Sciences.

[ref-68] Schaeck M, Reyes-López FE, Vallejos-Vidal E, Van Cleemput J, Duchateau L, Van den BW, Tort L, Decostere A (2017). Cellular and transcriptomic response to treatment with the probiotic candidate *Vibrio lentus* in gnotobiotic sea bass (*Dicentrarchus labrax*) larvae. Fish & Shellfish Immunology.

[ref-69] Schwarzenbach RP, Escher BI, Fenner K, Hofstetter TB, Johnson CA, von Gunten U, Wehrli B (2006). The challenge of micropollutants in aquatic systems. Science.

[ref-70] Shen X, Miao J, Wan Q, Wang S, Li M, Pu F, Wang G, Qian W, Yu Q, Marotta F, He F (2018). Possible correlation between gut microbiota and immunity among healthy middle-aged and elderly people in southwest China. Gut Pathogens.

[ref-71] Simmons DB, Benskin JP, Cosgrove JR, Duncker BP, Ekman DR, Martyniuk CJ, Sherry JP (2015). Omics for aquatic ecotoxicology: control of extraneous variability to enhance the analysis of environmental effects. Environmental Toxicology and Chemistry.

[ref-72] Singh BK, Thakur K, Kumari H, Mahajan D, Sharma D, Sharma AK, Kumar S, Singh B, Pankaj PP, Kumar R (2025). A review on comparative analysis of marine and freshwater fish gut microbiomes: insights into environmental impact on gut microbiota. FEMS Microbiology Ecology.

[ref-73] Soh M, Tay YC, Lee CS, Low A, Orban L, Jaafar Z, Seedorf H (2024). The intestinal digesta microbiota of tropical marine fish is largely uncultured and distinct from surrounding water microbiota. NPJ Biofilms and Microbiomes.

[ref-74] Sorek R, Cossart P (2010). Prokaryotic transcriptomics: a new view on regulation, physiology and pathogenicity. Nature Reviews Genetics.

[ref-75] Spilsbury F, Foysal MJ, Tay A, Gagnon MM (2022). Gut microbiome as a potential biomarker in fish: dietary exposure to petroleum hydrocarbons and metals, metabolic functions and cytokine expression in Juvenile *Lates calcarifer*. Frontiers in Microbiology.

[ref-76] Sumithra TG, Sharma KSR, Gangadharan S, Suresh G, Prasad V, Amala PV, Sayooj P, Gop AP, Anil MK, Patil PK, Achamveetil G (2022). Dysbiosis and restoration dynamics of the gut microbiome following therapeutic exposure to florfenicol in snubnose pompano (*Trachinotus blochii*) to aid in sustainable aquaculture production strategies. Frontiers in Microbiology.

[ref-77] Sumithra TG, Sharma SRK, Suresh G, Suja G, Prasad V, Gop AP, Patil PK, Gopalakrishnan A (2024). Gut microbes of a high-value marine fish, snubnose pompano (*Trachinotus blochii*) are resilient to therapeutic dosing of oxytetracycline. Scientific Reports.

[ref-78] Tarigan M, Raji S, Al-Fatesh H, Czermak P, Ebrahimi M (2025). The occurrence of micropollutants in the aquatic environment and technologies for their removal. Processes.

[ref-79] Thompson RC, Olsen Y, Mitchell RP, Davis A, Rowland SJ, John AWG, McGonigle D, Russell AE (2004). Lost at sea: where is all the plastic?. Science.

[ref-80] Travers-Trolet M, van Denderen PD, Deniau C, Gascuel D, Lobry J, Trueman C, Cabral H, Lepage M, Lobry J, Le Pape O (2025). Chapter 13—the role of fish in marine food webs. Ecology of Marine Fish.

[ref-81] Tripathi A, Debelius J, Brenner DA, Karin M, Loomba R, Schnabl B, Knight R (2018). The gut-liver axis and the intersection with the microbiome. Nature Reviews Gastroenterology & Hepatology.

[ref-82] Ugwu K, Herrera A, Gómez M (2021). Microplastics in marine biota: a review. Marine Pollution Bulletin.

[ref-83] Uniacke-Lowe S, Stanton C, Hill C, Ross RP (2024). The marine fish gut microbiome as a source of novel bacteriocins. Microorganisms.

[ref-84] Walter JM, Bagi A, Pampanin DM (2019). Insights into the potential of the atlantic cod gut microbiome as biomarker of oil contamination in the marine environment. Microorganisms.

[ref-85] Wang Y, Hamid N, Deng S, Jia P-P, Pei D-S (2020). Individual and combined toxicogenetic effects of microplastics and heavy metals (Cd, Pb, and Zn) perturb gut microbiota homeostasis and gonadal development in marine medaka (*Oryzias melastigma*). Journal of Hazardous Materials.

[ref-86] Wang M, Qin Y, Liu Y, Yang H, Wang J, Ru S, Cui P (2023). Short-term exposure to enrofloxacin causes hepatic metabolism disorder associated with intestinal flora dysbiosis in adult marine medaka (*Oryzias melastigma*). Marine Pollution Bulletin.

[ref-87] Wang AR, Ran C, Ringo E, Zhou ZG (2018). Progress in fish gastrointestinal microbiota research. Reviews in Aquaculture.

[ref-88] Wang A, Zhang Z, Ding Q, Yang Y, Bindelle J, Ran C, Zhou Z (2021). Intestinal cetobacterium and acetate modify glucose homeostasis via parasympathetic activation in zebrafish. Gut Microbes.

[ref-89] Wen S, Yin X, Zhang Y, Diao X (2024). Chronic exposure to low concentrations of microplastics causing gut tissue damage but non-significant changes in the microbiota of marine medaka larvae (*Oryzias melastigma*). Marine Environmental Research.

[ref-90] Yaacob EN, Goethals J, Bajek A, Dierckens K, Bossier P, De Geest BG, Vanrompay D (2017). Preparation and characterization of alginate microparticles containing a model protein for oral administration in gnotobiotic European sea bass (*Dicentrarchus labrax*) larvae. Marine Biotechnology.

[ref-91] Yao FC, Jin CX, Liang H, Zhang Y, Gu Y, Song FB, Zhou Z, Sun JL, Luo J (2024). Microplastics weaken the digestion and absorption functions in the golden pompano (*Trachinotus blochii*) by affecting the intestinal structure, bacteria and metabolites. Chemosphere.

[ref-92] Yu LL, Xia W, Du H (2024). The toxic effects of petroleum pollutants to microalgae in marine environment. Marine Pollution Bulletin.

[ref-93] Zhai Q, Yu L, Li T, Zhu J, Zhang C, Zhao J, Zhang H, Chen W (2017). Effect of dietary probiotic supplementation on intestinal microbiota and physiological conditions of Nile tilapia (*Oreochromis niloticus*) under waterborne cadmium exposure. Antonie Van Leeuwenhoek.

[ref-94] Zhang YT, Chen R, Wang F, Huang Z, He S, Chen J, Mu J (2022). Potential involvement of the microbiota-gut-brain axis in the neurotoxicity of triphenyl phosphate (TPhP) in the marine medaka (*Oryzias melastigma*) larvae. Science of the Total Environment.

[ref-95] Zhou L, Limbu SM, Shen M, Zhai W, Qiao F, He A, Du Z-Y, Zhang M (2018). Environmental concentrations of antibiotics impair zebrafish gut health. Environmental Pollution.

[ref-96] Zhou YX, Zhang ZS (1989). Methods for aquatic organism toxicity testing.

